# Six Genes Associated with the Clinical Phenotypes of Individuals with Deficient and Proficient DNA Repair

**Published:** 2008-02-10

**Authors:** Tobias Gremmel, Susanne Wild, Winfried Schuller, Viola Kürten, Klaus Dietz, Jean Krutmann, Mark Berneburg

**Affiliations:** 1 Institut für Umweltmedizinische Forschung at the Heinrich-Heine-University gGmbH, Auf’m Hennekamp 50, D-40225 Düsseldorf, Germany; 2 Molecular Oncology and Aging, Department of Dermatology, Eberhard-Karls-University, Tübingen, Germany; 3 Department of Dermatology, Heinrich-Heine-University, Moorenstr. 5, D-40225 Düsseldorf, Germany; 4 Department of Medical Biometry, Eberhard-Karls-University, Tübingen, Germany

**Keywords:** array analysis, basal cell carcinoma, DNA repair, squamous cell carcinoma, skin cancer risk, xeroderma pigmentosum

## Abstract

Xeroderma pigmentosum (XP) is a genetic disorder characterised by hypo-/hyperpigmentation, increased sensitivity to ultraviolet (UV)-radiation and an up to 2000-fold increased skin cancer risk. Cells from XP-patients are defective in nucleotide excision repair (NER) responsible for repair of UV-induced DNA damage. This defect accounts for their mutator phenotype but does not predict their increased skin cancer risk. Therefore, we carried out array analysis to measure the expression of more than 1000 genes after UVB-irradiation in XP cells from three complementation groups with different clinical severity (XP-A, XP-D, XP-F) as well as from patients with normal DNA repair but increased skin cancer risk (≥2 basal or squamous cell carcinoma at age <40yrs). Of 144 genes investigated, 20 showed differential expression with p < 0.05 after irradiation of cells with 100 mJ/cm^2^ of UVB. A subset of six genes showed a direct association of expression levels with clinical severity of XP in genes affecting carcinogenesis relevant pathways. Genes identified in XP cells could be confirmed in cells from patients with no known DNA repair defects but increased skin cancer risk. Thus, it is possible to identify a small gene subset associated with clinical severity of XP patients also applicable to individuals with no known DNA repair defects.

## Introduction

Xeroderma pigmentosum (XP) is an autosomal recessive disorder with an estimated prevalence of 1:10^6^ worldwide. Clinically, it is characterised by hypo- and hyperpigmentation, telangiectasia and xerosis of the skin.^1^–^3^ Furthermore, patients with XP show photosensitivity and a risk to develop skin cancer that is increased up to 2000-fold.^4^ Cells from patients with XP are defective in nucleotide excision repair (NER), a mechanism responsible for the repair of helix distorting DNA damage including damage induced by ultraviolet (UV) radiation.^5^–^7^ While it is clear that defective DNA repair of UV-induced photoproducts leads to mutagenic photoproducts, this alone does not account for the increased skin cancer risk in these patients.^8^ In line with this notion, it is known that carcinogenesis, including UV-induced skin carcinogenesis, involves several processes such as p53 signalling, apoptosis, cell cycle control, DNA repair and immunosurveillance^9^,^10^ and it has been shown that e.g. DNA repair, immunology and apoptosis are in bidirectional cross-talk.^11^,^12^ In support of the notion that photocarcinogenesis in XP patients may involve multiple factors, we have shown previously, that the absence of an increased skin cancer risk in two different DNA repair deficiency syndromes, namely Trichothiodystrophy (TTD) and Cockayne syndrome (CS) is not associated with the DNA repair defect of these syndromes.^13^

Different gene expression profiles for skin carcinogenesis have been published.^14^–^16^ Furthermore, an elegant study by da Costa et al. has differentiated the transcriptional profile in cells expressing the XPB/CS or XPB/TTD allele after UVC-irradiation.^17^ However, thus far results derived from gene expression profiling in DNA repair deficient cells after physiologically relevant doses of UVB-irradiation have not been attempted to be transferred to the DNA repair proficient background to identify a defined set of genes with functional relevance for skin carcinogenesis and possible prognostic value.

Investigation of differential gene expression in cells from patients with three different complementation groups of XP, characterized by distinct severities of their clinical phenotypes identified a subset of genes with functions in processes relevant for UV-induced skin carcinogenesis. Genes identified could also be detected in cells from patients with no known defects in DNA repair but increased skin cancer risk at a magnitude that continued tendencies discovered in DNA repair deficient cells. The detection of this gene-subset may allow further work to identify factors determining the lifetime skin cancer risk of an individual in the normal population.

## Materials and Methods

### Cell culture

Fibroblast cultures were established from skin biopsies taken from patients with informed consent as approved by the ethics committee of the University of Düsseldorf, Germany (Nr.1480). Normal fibroblasts were derived from non-sunexposed skin of normal individuals of skin phototype II-III and estimated lifetime sun exposure of a caucasian individual in the northern hemisphere without outdoor occupation, and these were matched to the ages of the investigated patients. Xeroderma pigmentosum cell lines were XP19BR (XP-A), XP15BR (XP-A, complex splice mutation (personal communication A. Lehmann)), XP16BR (XP-D, amino acid change: R683W), XP2DF (XP-D), XP24BR (XP-F) and XP4DF (XP-F). These cells were kindly provided by Alan Lehmann from the Genome Stability Center of the University of Sussex Falmer Brighton, Great Britain with the exception of XP2DF and XP4DF which were generated and characterized by our own group. Details of the cell lines employed are given in Table 1. Patients suffering from complementation group XP-D did not show any features of trichothiodystrophy or Cockayne syndrome and patients with XP-F clinically showed the mildest phenotype as well as one XP-F patient showing the highest age, 66 years. Patients with ≥2 basal or squamous cell carcinoma were included in the study. Six of these patients were female and one male. Ages at enrolment ranged between 18 and 39. Numbers of tumours prior to enrolment ranged between 2 and 10. Primary fibroblasts were cultured in MEM (PAA Laboratories, Pasching, Austria) supplemented with 15% FCS (Peribo Science, Bonn, Germany) and kept at 37 °C in a humidified atmosphere with 5% CO_2_. For culture and irradiation cells were kept in 10-cm culture dishes and grown to confluency to avoid cell cycle influence.

### Unscheduled DNA synthesis (UDS)

Measurement of DNA repair capacity was carried out similarly to the method described by Lehmann et al.^18^ In our experiments radioactive measurement of 3-H-thymidine incorporation was replaced by peroxidase based fluorescence measurement of 5-Bromo-2′-deoxy-uridine (BrdU) incorporation and the experimental procedure was carried out employing the Cell Proliferation Biotrak ELISA kit, version 2 (Amersham, Freiburg, Germany), according to the protocol. Briefly, fibroblasts were plated at 1 × 10^5^ in triplicates overnight followed by three days of serum starvation with medium containing 0.5% FCS. Subsequently, cells were exposed to 10 mM hydroxyurea for 30 min, washed, and then irradiated with different doses of UVC. Immediately after irradiation, cells were incubated with labelling solution including 10 μM BrdU for 3 hours. Labelling solution was removed followed by fixation (room temperature, 30 min), staining with peroxidase-labelled monoclonal anti-BrdU antibody and fluorescence detection by an Emax ELISA reader (Molecular Devices, Workingham, Great Britain).

### UVB-Irradiation

Irradiation was carried out as described previously.^19^,^20^ Briefly, lids were removed from tissue culture dishes, cells were washed with PBS once and irradiated with FS20 tubes (Westinghouse Electric, Pittsburgh, PA), which are known to emit primarily in the UVB range, with a tube-to-target distance of 22 cm and a UVB-dose of 100 mJ/cm^2^. The UVB output was monitored by means of an IL1700 research radiometer and SED 240 UVB photodetector (International Light, Newburyport, MA, USA).

### Array analysis

For array analysis, cells were harvested after an incubation time of 4 hours following UVB- or sham-irradiation and total RNA was extracted by employing the RNA Minikit (Qiagen, Hilden, Germany). Generation of cDNA was carried out employing a gene specific primer mix for Atlas^R^ Human 1.2 arrays (Clontech, Heidelberg, Germany) containing 1,185 known human genes according to the manufacturers protocol. Amplified cDNA was ^32^P-labelled and hybridised to nylon array membranes for 24 hours followed by Phosphorimager^®^ (Molecular Dynamics, Heidelberg, Germany) analysis of signal intensities. Quantification was carried out with AtlasImage^®^ Software normalizing values of cells from XP-patients and from patients with normal DNA repair but increased skin cancer risk to values from normal control cells, thus comparing normalised ratios of expression levels.

### Real time RT-PCR

Real time RT-PCR was carried out by using the commercially available TaqMan Gene Expression Assay by Applied Biosystems containing 250 nM final concentration FAM™ dye-labeled TaqMan^®^ MGB probe, 900 nm final concentration unlabeled PCR primer oligonucleotides as well as PCR reagents to facilitate real time RT-PCR for inter-leukin 2 receptor alpha, according to the manufacturers protocol. PCR reactions were set up in triplicates and performed three times separately for XP-patients as well as DNA repair proficient patients. Data are presented as means ± SD.

### Statistical analysis

For description of the repair capacity (UDS) by non-linear least squares we fitted the following model to the data:

y=a[1-exp(-bx)],

where *y* is the UDS in %, *x* is the UVC-dose in J/cm^2^, *a* is the asymptotic UDS for large doses and *b* is the rate of approach to the asymptotic value. The inverse of *b* multiplied by natural log of 2 (=0.693) equals the dose D50 at which 50% of the asymptote is reached. For the 7 normals/patients matched pairs we tested whether the means of the difference of the parameters *a* and *b* are equal to zero employing one-sample t-test.

Three separate experiments were carried out for each cell line, expression levels of investigated cells were normalized to values from aged matched normal controls and means were generated from this data. Each described set of data and genes determined by array analysis were included in further statistical analysis for which at least two data points were available. In XP cells, for both cells from each complementation group this minimum of two data points had to be available. Gene expression of XP cells was compared with that of patients by Student’s t-test for all genes included in the subset of 144 genes by using the statistical software package JMP (www.jmp.com). 20 of these genes showed a p-value <0.05 as shown in Table 2. In order to achieve normally distributed variates logarithmic transformations of the original data were used. Statistical significance was determined by calculating the q-values for each gene on the basis of the corresponding p values based on the false detection rate (FDR) as developed for array analysis.^21^ We followed exactly the method proposed by Storey and Tibshirani except that we replaced the cubic spline by an exponential function in determining the proportion of genes with no effect. In our data set, this method revealed p-values smaller than 0.0025 to be statistically significant (as denoted by an asterisk in Table 2). Of the genes with p < 0.05 six showed a direct association of gene expression with the clinical severity of XP complementation groups. These associations were illustrated by the 95% confidence intervals of the complementation group specific means as calculated by a one-way analysis of variance (Fig. 3).

## Results

### Confirmation of UDS levels in employed cells

**Figure 1:**
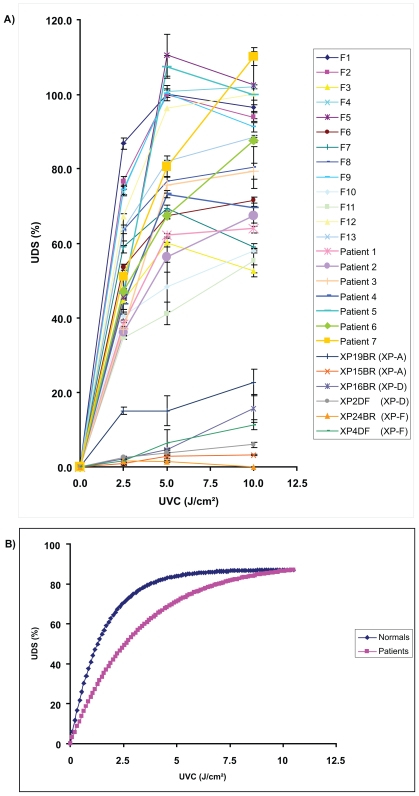
Measurement of UDS in cells from patients with at least 2 skin tumours before the age of 40 **A**) Assessment of UDS by BrdU-incorporation for fibroblasts from normal individuals, patients with increased skin cancer risk and XP-patients. Cells from patients with increased skin cancer and XP were age matched to normal fibroblasts e.g. F1 is matched to Patient 1 and F8 to XP19BR. All seven patients show normal levels of UDS with values of 50% of normal control cells or above at 10 J/cm^2^. Cells from patients of XP complementation groups XP-A, XP-D and XP-F served as negative controls with values staying below 30% of normal control cells. Values for UDS-associated BrdU-incorporation are shown in % relative to values of control cells ± SD. **B**) Mean values of UDS. Data are given as means of normal and patient cells. The maximum value of UDS repair capacity (asymptotic value) at dose 10 J/cm^2^ is the same in normals and patients but normal cells reach this value already at lower doses.

To ensure deficient DNA repair in XP cells as well as normal repair in cells derived from patients with increased skin cancer risk UDS was carried out in the cells employed (Fig. 1a). For XP cells UDS was abnormal as published previously. For fibroblasts from normals and patients with increased skin cancer risk, the asymptotic value of UDS for large doses (10 J/cm^2^) did not differ and was within the normal range (means ± SE: 87.1 ± 8.6% and 90.5 ± 8.3%, respectively). The D50 for fibroblasts from normals and patients were 1.04 and 2.24 respectively (Fig. 1b). The ratio of the two D50 values was 2.06 (95% confidence interval 1.24 to 3.41; p = 0.0175). For pairs of patients the power for this observed difference was 77%. In order to detect a difference of 20% in the asymptotic UDS value one would need 26 matched patients pairs with a power of 80% and a significance level of 5%.

### Identification of a defined subset comprising genes with differential expression after UVB with p-values<0.05

**Figure 2:**
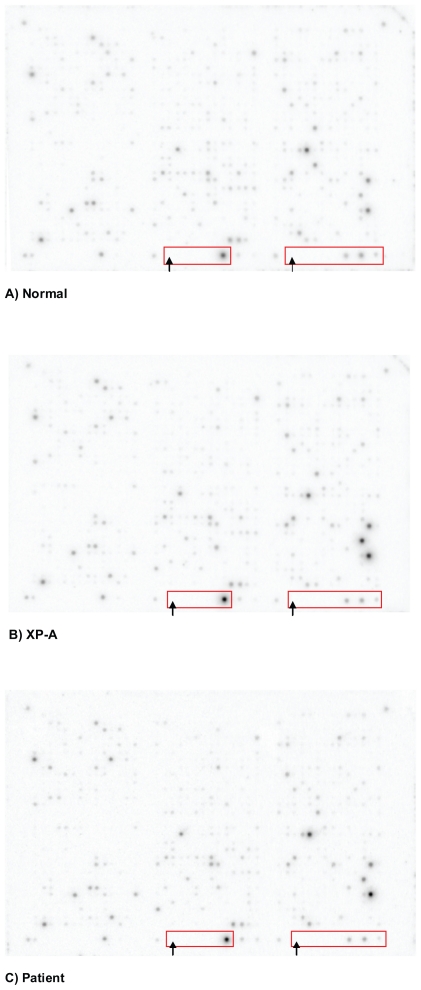
Representative array membranes of unirradiated fibroblasts as seen after 24 h exposure of nylon membranes to Phosphorimager^®^ screens **A**) Normal cells **B**) Cells from patient with XP-A; **C**) Cells from patient with normal DNA repair but increased skin cancer risk. No significant differences between arrays of different cells without irradiation were detected. Negative control DNA and housekeeping genes are circled at the bottom of each array. They are from left to right: lambda DNA (left arrow), liver glyceraldehyde 3-phosphate dehydrogenase (GAPDH), pUC18 DNA (right arrow), cytoplasmic beta-actin, 23-kDa highly basic protein, 40S ribosomal protein S9.

Differential gene expression in cells from patients with XP complementation groups of different clinical severity compared to normal cells was measured by Atlas Human 1.2 Arrays after sham- or UVB-irradiation with 100 mJ/cm^2^ containing 1,185 known genes. The signal intensity for control housekeeping genes showed no variation between experiments, indicating comparable hybridization levels for all experiments (Fig. 2a, Array picture of normal cells; 2b, Array picture of XP cells). Detected levels of gene expression in sham irradiated cells did not show any patterns attributable to clinical severity or other parameters. However, when measuring expression levels in UVB-exposed cells, differential gene expression could be detected in 144 genes comprising adequate data sets. Of these 144 genes, a total of 20 genes showed a p-value<0.05 (Table 2). Most of the genes detected by array analysis could be classified in functionally relevant pathways such as DNA damage signalling and repair, cell cycle control, apoptosis, protooncogenes and tumour suppressor genes, transcription activators and basic transcription factors as well as cytoskeleton/motility proteins (Table 2). Of these 20 genes, determination of positive false detection rates (pFDR) controlling for multiple measurements as described by Storey et al.^21^ revealed a number of three genes (Insulin like growth factor, ubiquitin C, colony stimulating factor 1), denoted by an asterisk in Table 2, to be statistically significant in our data set.

### Association of gene expression levels with clinical severity of XP complementation groups

**Figure 3:**
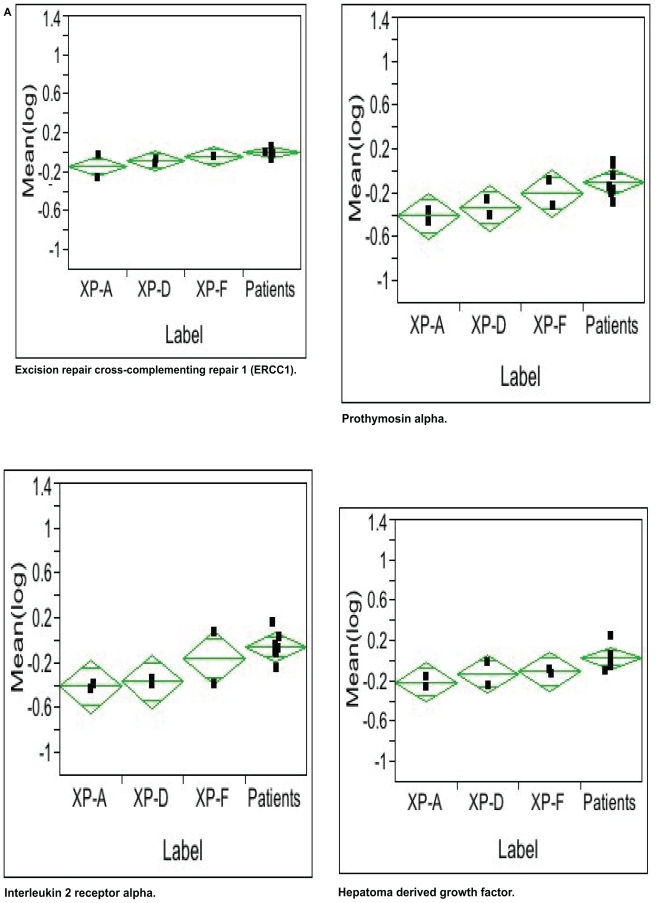

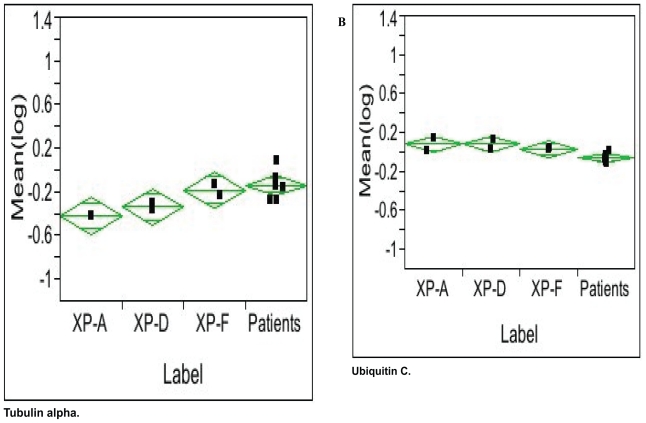
Identification of a defined subset of genes with association of gene expression level and clinical severity Gene expression levels of cells normalized to control cells are shown for XP-A (severe clinical phenotype), XP-D (intermediate clinical phenotype) and XP-F (milder clinical phenotype). Expression levels of cells from patients with normal DNA repair but increased skin cancer risk are shown next to the milder XP-F phenotype. **A**) Genes showing low levels for XP-A, intermediate levels for XP-D and high levels for XP-F. **B**) High level of gene expression in XP-A, intermediate level in XP-D and lower level in XP-F. Values for cells from XP-patients as well as values for patients with normal DNA repair but increased skin cancer risk were normalized to values from normal control fibroblasts and are given as natural logarithms of the ratios on the y-axis. The diamonds show the 95% confidence intervals (upper and lower line) of the corresponding means (central line).

When comparing clinical severity of XP symptoms with levels of gene expression after UVB-irradiation, a monotone association could be detected in six genes (Fig. 3). Of these six genes five (Excision repair cross complementing repair (ERCC)-1, interleukin 2 receptor alpha, prothymosin alpha, hepatoma derived growth factor, tubulin alpha) showed an increasing tendency of gene expression with a low expression level in complementation group XP-A, intermediate expression level in XP-D and a high expression level in XP-F (Fig. 3a). The sixth gene (ubiquitin C) showed a decreasing tendency of gene expression with high levels in XP-A, intermediate in XP-D and low levels of expression in XP-F, respectively (Fig. 3b). The six genes showing association with the clinical severity of the XP-complementation groups involve genes in functionally relevant pathways including DNA repair (ERCC-1), immunosurveillance (interleukin 2 receptor alpha), cell cycle control (prothymosin alpha), stress response (ubiquitin C), growth factors (hepatoma derived growth factor) as well as cytoskeleton/motility proteins (tubulin alpha) as shown in Table 2.

### Confirmation of results in XP cells by array analysis in cells from patient with no known defect in DNA repair but increased skin cancer risk

Genes with expression levels in association with the clinical severity of different XP complementation groups could be confirmed in cells from patients with normal UDS but increased skin cancer risk (Fig. 3). As with XP cells, array analysis showed comparable expression levels for housekeeping controls and no discernible patterns in sham-irradiated cells (Fig. 2c). In UVB-irradiated cells the previously detected 20 genes were also regulated with no association of clinical phenotype to either number or type of tumours or age of the patients (Table 3, bold; Fig. 3). Particularly, the tendency identified in cells from different XP complementation groups was continued in these cells in an extrapolating manner. In all six genes expression levels were next to the expression levels of the mildest XP complementation group, XP-F (Fig. 3).

### Confirmation of array data by real time RT-PCR

**Figure 4:**
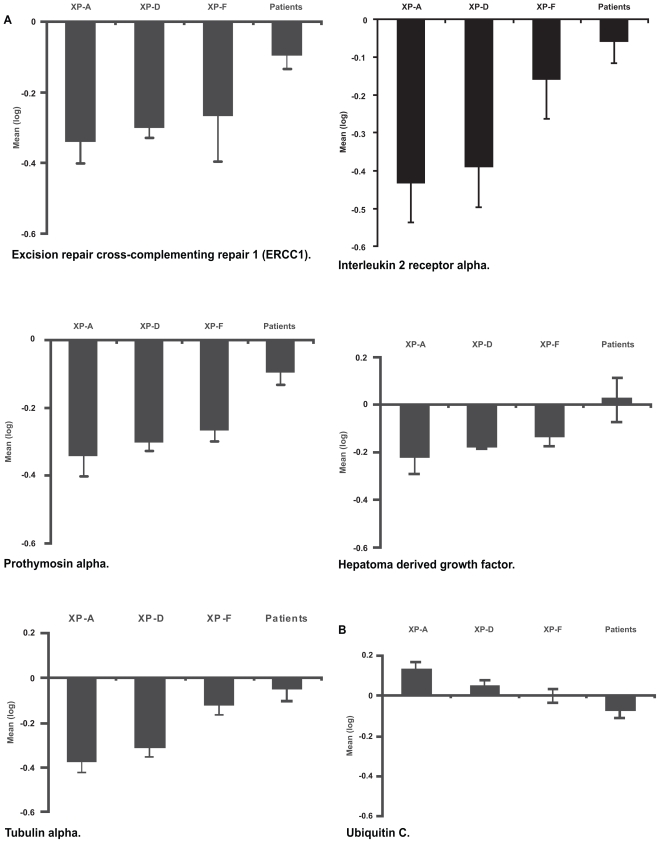
Real time RT-PCR for confirmation of data generated by array analysis Expression levels in two cell lines from patients with XP-A, XP-D, XP-F and all cells from individuals with no known defect in DNA repair but increased skin cancer risk (Patients). The logarithms of the data are presented as means +/− SD of three separate experiments. Expression levels determined by RT-PCR parallel levels detected by array analysis (compare Fig. 3).

The expression levels detected by array analysis could be confirmed by real time RT-PCR. Figure 4 shows gene expression levels for all genes identified by array analysis, thus confirming the data generated by array analysis (Fig. 4).

## Discussion

The risk of a certain individual to develop skin cancer is determined by genetic and environmental factors. The exact relative influence of each factor as well as the susceptibility of one individual to a given amount of environmental factors is currently unclear. These results indicate that it is possible to identify a defined subset of genes whose expression levels are associated with the clinical severity of different XP-complementation groups. Furthermore, the identified genes can be applied to a background with no known defects in DNA repair but increased skin cancer risk.

Genes identified by our analysis have been found by different approaches from other groups. Serewko et al. investigated the differential gene expression of epidermal cells during squamous cell carcinoma development^16^ and identified cell cycle genes, EGF receptors, calpains, growth factors, MAP kinases and insulin like growth factor receptors, also applicable to seven of the 20 genes identified in our study. Furthermore, a study by Dong et al. investigated metastatic murine squamous carcinoma cells by differential display^22^ where 3 of the six genes identified by us to be associated with the clinical phenotypes of patients were the same in that study. Genes found to be differentially expressed after UVB and statistically significant were also identified by further studies.^15^,^23^

We have previously shown that ICAM-1 expression is associated with the skin cancer risk in cells from patients with XP. It was unclear however, whether this holds true for cells from patients with normal DNA repair but increased skin cancer risk. In those studies UVB-mediated suppression of interferon-γ induced ICAM-1 expression was investigated. In the present study constitutive ICAM-1 expression was measured by array technology. This did not show any association of expression levels after UVB-irradiation with the clinical phenotypes of XP patients or patients with normal DNA repair. This is most likely due to differences of the experimental design, since it has been shown that in a number of cells such as keratinocytes and fibroblasts, constitutive ICAM-1 expression can not or only at higher doses be suppressed by UV-irradiation. It has to be noted that identification of differential gene expression by array analysis always implies the risk of false positive detection or spurious association even though we controlled for this by performing three independent experiments for two XP-cell lines and seven patients with normal DNA repair and statistical evaluation of positive false detection rate.^21^ Bearing all this in mind, however, these results may nevertheless indicate, that the investigation of a single gene e.g. ICAM-1, may be sufficient to distinguish between cancer prone XP and non-cancer prone TTD but it may not be sufficient to identify an increased skin cancer risk in individuals with no known defects in DNA repair capacity. In our present study, simultaneous measurement of 1,185 genes by array technology did reveal six genes that are indeed associated with the skin cancer risk of investigated individuals. This may indicate a higher sensitivity of array assessment as compared to measurement of any single gene, this being the case even in a background with no known defects in DNA repair.

In the present study the NER gene ERCC-1 was found to be associated with the clinical phenotypes of DNA repair deficient and proficient patients. Since it has been shown previously that expression of NER genes such as *XPC* and *XPE* can be transactivated by p53 after UV-radiation^30^ the present data confirm the notion that NER genes can be differentially expressed and that they may be associated with the clinical phenotype not only in XP patients but also in individuals with no known defect in DNA repair but increased skin cancer risk. Genes identified by a study investigating differential gene expression in cells with the XPB/CS or XPB/TTD allele^17^ also confirmed genes identified by our work (tubulin alpha, MHC-I, Insulin like growth factor) albeit this study employed UVC-irradiation.

Investigation of differential gene expression analysis has been carried out extensively previously with the intention to identify genes regulated by ultraviolet radiation or during skin carcinogenesis.^14^–^16^,^22^,^24^–^27^ In the present study this intention was only secondary. One of the strengths of array analysis is their application as a diagnostic technology.^28^,^29^ The present work identified a restricted and defined subset of genes generated from well established syndromes. These genes were then transferred to a background with no known defect in DNA repair. Further studies are necessary to evaluate the prognostic value of this gene subset in the normal population.

## Figures and Tables

**Table 1:** Details of cell lines used from patients with increased skin cancer and XP.

Patient	Group	Age	Biopsy skin site	Phototype	Sun exposure	Passage	Tumour
1		18 y	Non-sunexposed	III	Intermediate	8	BCC
2		21 y	Non-sunexposed	III	Intermediate	7	BCC
3		39 y	Non-sunexposed	II	Intermediate	2	BCC
4		29 y	Non-sunexposed	III	High	7	SCC
5		33 y	Non-sunexposed	II	Intermediate	6	BCC
6		36 y	Non-sunexposed	II	Intermediate	4	BCC
7		38 y	Sun-exposed	III	Intermediate	6	BCC
XP19BR	XP-A	15 y	Non-sunexposed	II–III	Low	2	
XP15BR	XP-A	0.3 y	Non-sunexposed	II–III	Low	6	
XP16BR	XP-D	1 y	Non-sunexposed	II–III	Low	6	
XP2DF	XP-D	21 y	Non-sunexposed	III	Inermediate	7	
XP24BR	XP-F	30 y	Non-sunexposed	I–II	Intermediate	4	
XP4DF	XP-F	66 y	Non-sunexposed	II	Intermediate	8	

Complementation group, age, body site of skin biopsy for fibroblast generation, skin phototype according to Fitzpatrick, estimated lifetime sun exposure, passage level of fibroblasts as well as the types of tumours are given where applicable. For estimated lifetime sun exposure low indicates close to no sun exposure at all due to XP-adequate protection, intermediate indicates sun exposure comparable to a caucasian individual in the northern hemisphere without occupational outdoor activity (includes XP patients applying no XP-specific sun protection) and high indicates sun exposure comparable to a caucasian individual in the northern hemisphere with occupational outdoor activity.

**Table 2:** Genes with differential gene expression following exposure to UVB.

Gene name	P	Code	Genbank	Locus link	Swissprot	Classification
**Excision repair cross-complementation 1 (ERCC1)**	**0,0289096**	**C01n**	**M13194**	**2067**	**P07992**	**DNA damage signaling/repair proteins and DNA ligases**
mutL (E. coli) homolog 1	0,0051168	C06n	U07418	4292	P40692	DNA damage signaling/repair proteins and DNA ligases, stress response proteins
CDC-like kinase1	0,0161107	A14i	L29222	1195	P49759	cell cycle-regulating kinases, nuclear proteins
prothymosin alpha	**0,0167602**	**A03l**	**M26708**	**5757**	**Q15249**	**other cell cycle proteins, oncogenes and tumour suppressors**
Ribosomal protein S19	0,0039686	A07l	M81757	6223	P39019	other cell cycle proteins, oncogenes and tumour suppressors
Mitogen-activated protein kinase kinase kinase 11	0,0025372	B04k	L32976	4296	Q16584	death kinases, intracellular kinase network members (non-receptor protein kinases)
Ubiquitin C*	**0,0008739**	**G11**	**M26880**	**7316**		**stress response proteins, cytoplasmic proteins, nuclear proteins**
v-jun avian sarcoma virus 17 homolog	0,0482950	A10c	J04111	3725	P05412	oncogenes and tumour suppressors, apoptosis associated proteins
Glucocorticoid receptor DNA binding factor 1	0,0364432	D07l	M73077	2909	Q14452	transcription activators and repressors, nuclear proteins;
EGF-response factor 1	0,0491504	D06j	X79067	677	Q07352	transcription activators and repressors, nuclear proteins;
Colony stimulating factor 1*	0,0010858	F03e	M37435	1435	P09603	growth factors, cytokines, and chemokines, extracellular secreted proteins
**Hepatoma-derived growth factor**	**0,0184383**	**F05e**	**D16431**	**3068**	**P51858**	**growth factors, cytokines, and chemokines, extracellular secreted proteins**
**Interleukin 2 receptor alpha**	**0,0153361**	**E01l**	**X01057**	**3559**	**P01589**	**interleukin and interferon receptors; plasma membrane proteins;**
Interleukin 1 beta	0,0068164	F10i	K02770	3553	P01584	interleukins and interferons; extracellular secreted proteins;
Ribosomal protein L13a	0,0371257	G45	X56932	23521	P40429	other immune system proteins
Major histocompatibility complex class I C	0,0305518	G31	M11886	3107	P10321	major histocompatibility complex; plasma membrane proteins;
**Tubulin alpha**	**0,0246099**	**G29**	**K00558**	**10376**	**P04687**	**cytoskeleton/motility proteins, cytoplasmic proteins, cytoskeletal proteins**
Calpain 2 (m/II) large subunit	0,0390684	C14h	M23254	824	P17655	calpains, cysteine proteases, cytoplasmic proteins
Insulin-like growth factor binding protein 3*	0,0002142	F12h	M31159	3486	P17936	hormones, extracellular secreted proteins
Heat shock 27kD protein 1	0,0381607	F05b	X54079	3315	P04792	heat shock proteins; cytoplasmic proteins;

Genes are clustered according to their respective classification. Genes with correlation to clinical severity of XP complementation groups as well as patients with normal DNA repair and increased skin cancer risk are shown in bold. P-values of respective genes are given as generated by Student’s t test and only genes with a p-value <0.05 are listed.

**Table 3:** Expression levels of differentially expressed genes.

Gene name	p	Code	XP-A	XP-D	XP-F	Patients
**Excision repair cross-compl. repair 1 (ERCC1)**	**0,0289096**	**C01n**	**−0.143**	**−0.090**	**−0.045**	**−0.001**
mutL (E. coli) homolog 1	0,0051168	C06n	−0.110	−0.159	−0.123	0.041
CDC-like kinase1	0,0161107	A14i	−0.165	−0.313	−0.140	0.054
**Prothymosin, alpha (gene sequence 28)**	**0,0167602**	**A03l**	**−0.411**	**−0.335**	**−0.203**	**−0.105**
Ribosomal protein S19	0,0039686	A07l	−0.084	−0.096	−0.023	0.095
Mitogen-activated protein kinase kinase kinase 11	0,0025372	B04k	−0.093	−0.233	−0.147	0.059
Ubiquitin C*	**0,0008739**	**G11**	**0.080**	**0.079**	**0.030**	**−0.064**
v-jun avian sarcoma virus 17 homolog	0,0482950	A10c	−0.016	−0.173	−0.037	0.081
Glucocorticoid receptor DNA binding factor 1	0,0364432	D07l	0.006	0.074	0.093	−0.043
EGF-response factor 1	0,0491504	D06j	0.123	0.236	0.427	0.050
Colony stimulating factor 1*	0,0010858	F03e	−0.051	−0.074	−0.062	0.098
**Hepatoma-derived growth factor**	**0,0184383**	**F05e**	**−0.211**	**−0.133**	**−0.109**	**0.026**
**Interleukin 2 receptor, alpha**	**0,0153361**	**E01l**	**−0.412**	**−0.369**	**−0.157**	**−0.050**
Interleukin 1, beta	0,0068164	F10i	−0.173	−0.128	−0.131	−0.046
Ribosomal protein L13a	0,0371257	G45	−0.163	−0.248	−0.156	−0.015
Major histocompatibility complex, class I, C	0,0305518	G31	−0.479	−0.233	−0.320	−0.039
**Tubulin alpha**	**0,0246099**	**G29**	**−0.418**	**−0.338**	**−0.182**	**−0.139**
Calpain 2, (m/II) large subunit	0,0390684	C14h	−0.135	−0.059	−0.062	0.023
Insulin-like growth factor binding protein 3*	0,0002142	F12h	1.045	1.253	1.220	0.389
Heat shock 27kD protein 1	0,0381607	F05b	−0.115	0.053	−0.036	0.096

Expression levels of genes are shown for patients with XP-A, XP-D, XP-F and patients with normal DNA repair but increased skin cancer risk. Genes with correlation to clinical severity of XP complementation groups as well as patients with normal DNA repair and increased skin cancer risk are shown in bold. Data is presented as means of three separate experiments from two cell lines of XP patients and seven patients with increased skin cancer risk normalized to age matched normal cells. P-values of respective genes are given as generated by Student’s t test and only genes with a p-value <0.05 are listed

## Abbreviations

BCC: Basal cell carcinoma
CS: Cockayne Syndrome
CPD: Cyclobutane-pyrimidine dimer
NER: Nucleotide excision repair
SCC: Squamous cell carcinoma
UDS: Unscheduled DNA-synthesis
TTD: Trichothiodystrophy
UV: Ultraviolet
XP: Xeroderma pigmentosum

## References

[1] Lehmann AR Jaspers NG Gatti RA 1989 Fourth International Workshop on Ataxia-Telangiectasia Cancer Res 49 6162 3 2676158

[2] Berneburg M Lehmann AR 2001 Xeroderma pigmentosum and related disorders: defects in DNA repair and transcription Adv. Genet 43 71 102 11037299 10.1016/s0065-2660(01)43004-5

[3] de Laat WL Jaspers NG Hoeijmakers JH 1999 Molecular mechanism of nucleotide excision repair Genes Dev 13 768 85 10197977 10.1101/gad.13.7.768

[4] Kraemer KH Lee MM Scotto J 1987 Xeroderma pigmentosum. Cutaneous, ocular, and neurologic abnormalities in 830 published cases Arch. Dermatol 123 241 50 3545087 10.1001/archderm.123.2.241

[5] Daya-Grosjean L Sarasin A 2005 The role of UV induced lesions in skin carcinogenesis: an overview of oncogene and tumour suppressor gene modifications in xeroderma pigmentosum skin tumours Mutat. Res 571 43 56 15748637 10.1016/j.mrfmmm.2004.11.013

[6] Volker M Mone MJ Karmakar P van Hoffen A Schul W Vermeulen W Hoeijmakers JH van Driel R Van Zeeland AA Mullenders LH 2001 Sequential assembly of the nucleotide excision repair factors in vivo Mol. Cell 8 213 24 11511374 10.1016/s1097-2765(01)00281-7

[7] Wood RD Mitchell M Lindahl T 2005 Human DNA repair genes 2005 Mutat Res10.1016/j.mrfmmm.2005.03.007 15922366

[8] Dubaele S Proietti DS Bienstock RJ Keriel A Stefanini M Van Houten B Egly JM 2003 Basal transcription defect discriminates between xeroderma pigmentosum and trichothiodystrophy in XPD patients Mol. Cell 11 1635 46 12820975 10.1016/s1097-2765(03)00182-5

[9] Berneburg M Krutmann J 2000 Photoimmunology, DNA repair and photocarcinogenesis J. Photochem. Photobiol. B 54 87 93 10836536 10.1016/s1011-1344(00)00024-5

[10] Hanahan D Weinberg RA 2000 The hallmarks of cancer Cell 100 57 70 10647931 10.1016/s0092-8674(00)81683-9

[11] Schwarz A Maeda A Kernebeck K van Steeg H Beissert S Schwarz T 2005 Prevention of UV radiation-induced immunosuppression by IL-12 is dependent on DNA repair J. Exp. Med 201 173 9 15657287 10.1084/jem.20041212 PMC2212783

[12] Schwarz A Stander S Berneburg M Bohm M Kulms D van Steeg H Grosse-Heitmeyer K Krutmann J Schwarz T 2002 Interleukin-12 suppresses ultraviolet radiation-induced apoptosis by inducing DNA repair Nat. Cell. Biol 4 26 31 11780128 10.1038/ncb717

[13] Ahrens C Grewe M Berneburg M Grether-Beck S Quilliet X Mezzina M Sarasin A Lehmann AR Arlett CF Krutmann J 1997 Photocarcinogenesis and inhibition of intercellular adhesion molecule 1 expression in cells of DNA-repair-defective individuals Proc. Natl. Acad. Sci. U.S.A 94 6837 41 9192652 10.1073/pnas.94.13.6837 PMC21245

[14] Howell BG Wang B Freed I Mamelak AJ Watanabe H Sauder DN 2004 Microarray analysis of UVB-regulated genes in keratinocytes: downregulation of angiogenesis inhibitor thrombospondin-1 J. Dermatol. Sci 34 185 194 15113588 10.1016/j.jdermsci.2004.01.004

[15] Izzotti A Cartiglia C Longobardi M Balansky RM D’Agostini F Lubet RA De Flora S 2004 Alterations of gene expression in skin and lung of mice exposed to light and cigarette smoke FASEB. J 18 1559 61 15289447 10.1096/fj.04-1877fje

[16] Serewko MM Popa C Dahler AL Smith L Strutton GM Coman W Dicker AJ Saunders NA 2002 Alterations in gene expression and activity during squamous cell carcinoma development Cancer Res 62 3759 65 12097286

[17] da Costa RM Riou L Paquola A Menck CF Sarasin A 2005 Transcriptional profiles of unirradiated or UV-irradiated human cells expressing either the cancer-prone XPB/CS allele or the non-cancer-prone XPB/TTD allele Oncogene 24 1359 74 15608684 10.1038/sj.onc.1208288

[18] Lehmann AR Stevens S 1980 A rapid procedure for measurement of DNA repair in human fibroblasts and for complementation analysis of xeroderma pigmentosum cells Mutat. Res 69 177 190 6987495 10.1016/0027-5107(80)90187-6

[19] Berneburg M Lowe JE Nardo T Araujo S Fousteri MI Green MH Krutmann J Wood RD Stefanini M Lehmann AR 2000 UV damage causes uncontrolled DNA breakage in cells from patients with combined features of XP-D and Cockayne syndrome EMBO J 19 1157 66 10698956 10.1093/emboj/19.5.1157 PMC305654

[20] Berneburg M Clingen PH Harcourt SA Lowe JE Taylor EM Green MH Krutmann J Arlett CF Lehmann AR 2000 The cancer-free phenotype in trichothiodystrophy is unrelated to its repair defect Cancer Res 60 431 8 10667598

[21] Storey JD Tibshirani R 2003 Statistical Significance For Genomewide Studies Proc. Natl. Acad. Sci. U.S.A 100 9440 9445 12883005 10.1073/pnas.1530509100 PMC170937

[22] Dong G Loukinova E Chen Z Gangi L Chanturita TI Liu ET Van Waes C 2001 Molecular profiling of transformed and metastatic murine squamous carcinoma cells by differential display and cDNA microarray reveals altered expression of multiple genes related to growth, apoptosis, angiogenesis, and the NF-kappaB signal pathway Cancer Res 61 4797 808 11406555

[23] Dooley TP Reddy SP Wilborn TW Davis RL 2003 Biomarkers of human cutaneous squamous cell carcinoma from tissues and cell lines identified by DNA microarrays and qRT-PCR Biochem. Biophys. Res. Commun 306 1026 36 12821146 10.1016/s0006-291x(03)01099-4

[24] Brosche M Schuler MA Kalbina I Connor L Strid A 2002 Gene regulation by low level UV-B. radiation: identification by DNA array analysis Photochem. Photobiol. Sci 1 656 64 12665302 10.1039/b202659g

[25] Clark EA Golub TR Lander ES Hynes RO 2000 Genomic analysis of metastasis reveals an essential role for RhoC Nature 406 532 5 10952316 10.1038/35020106

[26] Davies H Bignell GR Cox C Stephens P Edkins S Clegg S Teague J Woffendin H Garnett MJ Bottomley W Davis N Dicks E Ewing R Floyd Y Gray K Hall S Hawes R Hughes J Kosmidou V Menzies A Mould C Parker A Stevens C Watt S Hooper S Wilson R Jayatilake H Gusterson BA Cooper C Shipley J Hargrave D Pritchard-Jones K Maitland N Chenevix-Trench G Riggins GJ Bigner DD Palmieri G Cossu A Flanagan A Nicholson A Ho JW Leung SY Yuen ST Weber BL Seigler HF Darrow TL Paterson H Marais R Marshall CJ Wooster R Stratton MR Futreal PA 2002 Mutations of the BRAF gene in human cancer Nature 417 949 54 12068308 10.1038/nature00766

[27] Loercher A Lee TL Ricker JL Howard A Geoghegen J Chen Z Sunwoo JB Sitcheran R Chuang EY Mitchell JB Baldwin AS Jr Van Waes C 2004 Nuclear factor-kappaB is an important modulator of the altered gene expression profile and malignant phenotype in squamous cell carcinoma Cancer Res 64 6511 23 15374962 10.1158/0008-5472.CAN-04-0852

[28] Marx J 2000 Medicine. DNA arrays reveal cancer in its many forms Science 289 1670 72 11001727 10.1126/science.289.5485.1670

[29] Sawiris GP Sherman-Baust CA Becker KG Cheadle C Teichberg D Morin PJ 2002 Development of a highly specialized cDNA array for the study and diagnosis of epithelial ovarian cancer Cancer Res 62 2923 28 12019173

[30] Hanawalt PC Ford JM Loyd DR 2003 Functional characterization of global genomic DNA repair and its implications for cancer Mut. Res 544 107 114 14644313 10.1016/j.mrrev.2003.06.002

